# Nitrogen Doped Macroporous Carbon as Electrode Materials for High Capacity of Supercapacitor

**DOI:** 10.3390/polym9010002

**Published:** 2017-01-13

**Authors:** Yudong Li, Xianzhu Xu, Yanzhen He, Yanqiu Jiang, Kaifeng Lin

**Affiliations:** 1 MIIT Key Laboratory of Critical Materials Technology for New Energy Conversion and Storage, School of Chemistry and Chemical Engineering, Harbin Institute of Technology, Harbin 150001, China; lydlnn0000@163.com (Y.L.); xuxianzhu@hit.edu.cn (X.X.); 2 School of Chemistry and Chemical Engineering, Harbin Institute of Technology, Harbin 150001, China; heyanzhen2015@126.com

**Keywords:** N-doped, macropores, supercapacitors

## Abstract

Nitrogen doped carbon materials as electrodes of supercapacitors have attracted abundant attention. Herein, we demonstrated a method to synthesize N-doped macroporous carbon materials (NMC) with continuous channels and large size pores carbonized from polyaniline using multiporous silica beads as sacrificial templates to act as electrode materials in supercapacitors. By the nice carbonized process, i.e., pre-carbonization at 400 °C and then pyrolysis at 700/800/900/1000 °C, NMC replicas with high BET specific surface areas exhibit excellent stability and recyclability as well as superb capacitance behavior (~413 F⋅g^−1^) in alkaline electrolyte. This research may provide a method to synthesize macroporous materials with continuous channels and hierarchical pores to enhance the infiltration and mass transfer not only used as electrode, but also as catalyst somewhere micro- or mesopores do not work well.

## 1. Introduction

Supercapacitors (SCs) have been the hot topic in new energy research since the twentieth century [1,2]. Because of the vivid advantages of SCs, such as short operating time of charge and discharge, long cycle life, relatively high energy density, and power density [3,4,5,6,7], SCs are replacing environmentally harmful fuels in more and more fields. However, some shortcomings still need to be solved including the high cost, instability and low energy density; the key to solving these problems is the electrode materials, and excellent achievements have been achieved [8,9]. The application of N-doped carbon materials in oxygen reduction reaction (ORR) [10,11,12,13,14], Li-ion battery [15,16,17,18] and SCs [19,20,21,22,23,24] are widely researched. The electronic conductivity of electrode materials is enhanced and the specific capacitance is improved by the pseudocapacitance effect after the immigration of the N atoms [25,26,27]. At the same time, the infiltration of the materials increases due to the special characteristics of the N atom [28,29,30,31]. Generally, two methods to synthesize N-doped materials have been emphasized: in-situ doping and post-doping [32,33], in which the latter always uses NH_3_-post management or chemical activity to introduce the N atoms, leading to instable structure, low thermostability and reproducibility, and high toxicity. Therefore, the in-situ doping method is adopted regularly because it is easier and more controllable, although the BET specific surface area may be unacceptable [34,35,36]. Different morphologies of N-doped carbon materials such as nanotubes [37,38,39,40], graphene [41,42,43], mesoporous graphitic arrays [44,45], carbon xerogel [46], activated carbon powders and fibers [47,48] have been investigated, and they exhibit outstanding properties as electrodes in SCs. In view of the positive influence of the higher specific surface area on the properties, porous N-doped carbon materials permit a higher electrical double-layer capacitance, a higher infiltration degree between the electrode and the electrolyte and a higher mass transfer ability [49,50]. Porous N-doped carbon materials have been widely investigated as electrodes in SCs [51,52,53,54,55,56]. Micro- and mesoporous materials may expose a shortcoming in effective porosity caused by the low infiltration degree, leading to the low specific capacitance [57,58,59,60]. Perhaps due to the slight gap of the size between the electrolyte ions and the pore diameter, less attention has been paid to research on the macroporous or large-scale mesoporous N-doped carbon materials. Herein, we demonstrate a method to synthesize N-doped macroporous carbon materials (denoted as NMC) with continuous channels around 40–50 nm to increase the mass transfer and the movement of electrolyte ions and electrons, the NMC materials using pristine polyaniline (PANI) as C and N source are inverted from the multiporous silica bead inverting from the IRA-900 resin [61]. By the special carbonized technique, we successfully synthesized NMC materials possessing a high Brunauer-Emmett-Teller (BET) specific surface area jointly provided by the macropores, large-scale mesopores and the micropores connecting to each other. A perfect N-doped content is also obtained. These properties and structures can improve the mass transfer of the electrolyte and active charges by delivery through multilevel channels and pores and the infiltration degree is enhanced by the channels and pores as well as the N functional groups, thereby the capacitance increases.

## 2. Materials and Methods

### 2.1. Materials

Aniline (AR, 99%), FeCl_3_ and tetraethyl orthosilicate (TEOS) are purchased from Sinopharm Chemical Reagent Co., Ltd. (Shanghai, China). Amberlite IRA-900 resin in the chloride form is purchased from Alfa Aesar (Heysham, England). All chemicals are used without further purification after received.

### 2.2. Preparation of Multiporous SiO_2_ Beads Templates

**Figure 1:**
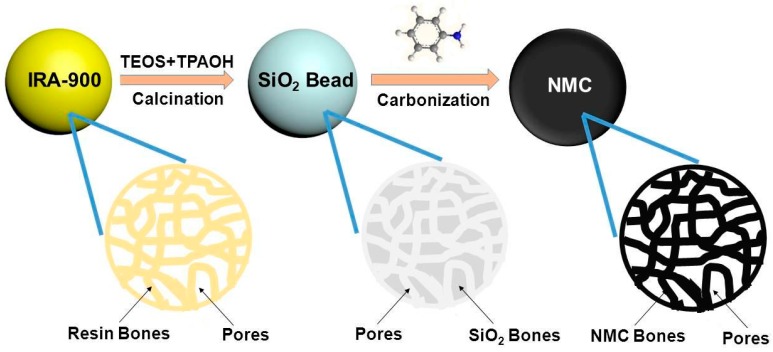
Schematic for the formation of multiporous SiO_2_ bead template and N-doped macroporous carbon (NMC) replicas.

The beads of multiporous SiO_2_ are obtained by a modified method according to the literature [61]. As shown in Figure 1, 2 mL of tetrapropyl-ammonium hydroxide (TPAOH) was mixed with 8 mL of H_2_O at 40 °C followed by addition of 5 mL of tetraethyl orthosilicate (TEOS). Next, Amberlite IRA-900 resin beads in the chloride format were added into the solution, and the weight ratio of solution/resin is 15:1. The mixture was stirred at 20 °C for 20 h and heated at 60 °C for 24 h in an autoclave. Then, the beads were separated and washed several times with deionized water, and dried at 60 °C. The beads were calcined in an oven at 550 °C for 6 h (heating rate of 1.5 °C/min) to remove all organic components and the white beads were obtained finally.

### 2.3. Preparation of N-Doped Macroporous Carbon Materials

The NMC materials in bead format were produced using multiporous silica beads as template and aniline as N-containing carbon precursor by heating the aniline under N_2_ flow. In a typical run, 1 g of the obtained SiO_2_ beads was mixed with aniline and HCl (2 M) at room temperature for at least 24 h. Then, 0.25 g FeCl_3_ aqueous solution (2 M) was added into the mixture and stirred for 24 h. Afterwards, a certain amount of ammonium persulfate (APS) was added into the mixture to polymerized sufficiently in the pores, the beads were separated and washed by ethanol and water several times until the filtrate become colorless, after that the beads are dried at 60 °C. Next, the beads impregnated with polyaniline were first thermally pre-carbonized under a gentle N_2_ flow at 400 °C at a rate of 2 °C/min for 2 h and then carbonized at a certain temperature (denoted as NMC-X, where X refers to the temperature (700, 800, 900 or 1000 °C)) for 1 h (heating rate of 2 °C/min). In order to remove SiO_2_ parts and Fe species, the thermally treated beads were washed with NH_4_HF_2_ aqueous solution and HCl solution, yielding NMC beads. The pristine PANI replica material was synthesized by removing the templates in the as-prepared bead mixed with PANI and silica bead.

### 2.4. Characterization

X-ray Diffraction (XRD) measurements are recorded on a Bruker D8 Advance diffractometer (Bruker Corporation, Karlsurhe, Germany) using Cu Ka radiation. Fourier Transform Infrared (FT-IR) spectra are measured utilizing Perkin Elmer 100 spectrometer (Perkin Corporation, Waltham, MA, USA). Scanning electron microscopy (SEM) and transmission electron microscopy (TEM) images are taken on a Hitachi S-4800 apparatus (Hitachi Ltd., Tokyo, Japan) and on a Topcon 002B transmission electron microscopy (ISI/Topcon Ltd., Tokyo, Japan), respectively. C/N molar ratios are determined by Elementar Analyzer VARIO Micro Cube (Elementar Corporation, Langenselbold, Germany). The isotherms of nitrogen adsorption–desorption are measured at liquid nitrogen temperature using a Micromeritics ASAP 2020 system (Micromeritics Corporation, Norcross (Atlanta), Norcross, GA, USA). The pore-size distribution is calculated using Barrett–Joyner–Halenda (BJH) model (Micromeritics Corporation, Norcross (Atlanta), Norcross, GA, USA).

### 2.5. Electrochemical Tests

A conventional three-electrode cell was adopt to examine the electro-chemical properties of NMC-X materials using a rotating disk electrode (RDE) in 6 M KOH solution as electrolyte on a CHI Electrochemical Station (Model 660D, Shanghai, China) at room temperature. Saturated calomel electrode (SCE) and platinum wire were employed as the counter and reference electrodes, respectively. In order to prepare the catalyst slurry, appropriate amounts of NMC materials were ultrasonically dispersed in an alcoholic solution containing 5 wt % Nafion for 1 h. The current voltage (CV) and galvanostatic charge/discharge at various scan rates and current densities were adopted to investigate the electrochemical capacitance properties of the prepared samples. The capacitance was calculated from the discharge CV data according to the following equation [62]:

(1) $$C=\frac{1}{\vartheta \left(V_{f}-V_{i}\right)}\int_{V_{i}}^{V_{f}}I\left(V\right)dV$$

where *C* is the capacitance from the electrodes, ϑ is the scan rate (V·s^−1^), *V*_f_ and *V*_i_ are the integration potential limits of the voltammetric curve, and *I*(*V*) is the discharge current (A). The mass capacitance *C*_m_ (F·g^−1^) was calculated based on the mass of samples, and, in three electrodes system, the mass loading for active materials is about 0.2 mg/cm^2^.

### 2.6. Fabrication of Supercapacitors in Two Electrodes System

Symmetric supercapacitor in a button cell sandwich form was assembled to evaluate the supercapacitive performance of NMC-800. The working electrode was produced by mixing the active material, carbon black and PTFE (60 wt % dispersion in water) in a weight ratio of 8:1:1 into a homogeneous slurry using an agate mortar and pestle. Then, the mixture was coated on to the current collector of nickel foam with a square length of 1 cm. After being dried at 120 °C for 6 h, the electrodes/collectors were fabricated in CR2032 stainless steel button cell with a porous polypropylene membrane and 6 M KOH aqueous solution as separator and electrolyte, respectively. The active material on each electrode was 2.0 mg. The CV tests of symmetric cell were performed in the voltage range of 0–1 V.

### 2.7. Electrochemical Calculations in Two Electrodes System

Specific capacitance of the electrode material (*C*_s_, F·g^−1^) was calculated [22] from CP discharge curve

$$C_{s}=\frac{2\times I_{\mathrm{cons}}\times \Delta t}{m\times \Delta V}$$

where *I*_cons_ is the constant current in discharging, *m* is the mass of active material on one electrode, Δ*t* is the discharge time, and Δ*V* is the voltage change during discharge (excluding the *IR* drop).

Energy density (*E*, W·h·kg^−1^) of the cell was calculated by

$$E=\frac{C_{s}\times V_{\max}{}^{2}}{2\times 4\times 3.6}$$

where *V*_max_ is the voltage at the beginning of discharge.

Average power density (*P*, W·kg^−1^) of the cell was obtained by

$$P=\frac{E\times 3600}{\Delta t}$$

## 3. Results and Discussion

### 3.1. Properties of NMC Replicas

**Figure 2:**
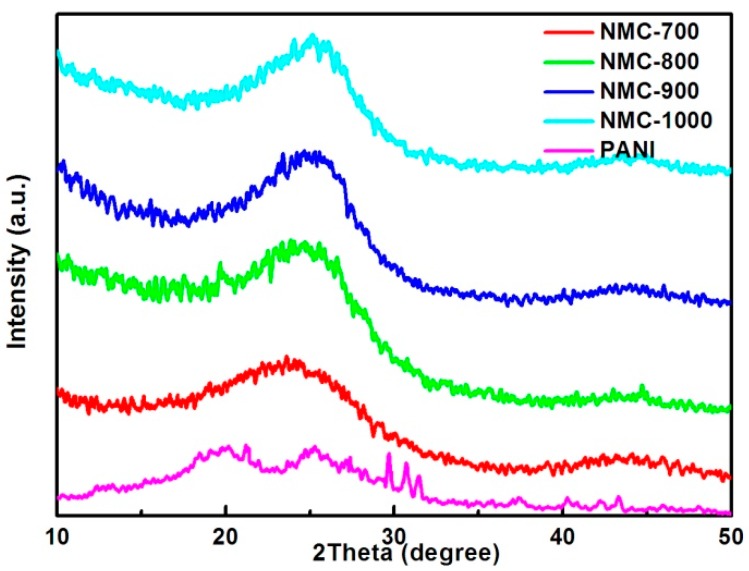
XRD patterns of NMC materials at different carbonized temperatures.

Figure 2 demonstrates the wide-angle XRD patterns for PANI and the NMC replicas in different carbonized temperatures. The PANI pattern shows two poignant peaks at about 2θ = 18° and 25°, which fit the characteristic peaks of PANI well [63]. However, after being carbonized in the NMC replica samples, a broad peak appears at around 2θ = 24° or 25°, which corresponds to the superimposed (002) diffraction of graphitic carbon and amorphous carbon formed by the conversion of PANI. The graphitic carbon is converted from the amorphous carbon by the catalysis of iron particles loaded in the matrix [64]. By the comparison of four carbonized materials, a slight difference appears, the (002) peak in NMC-800 to NMC-1000 shifting to a high angle compared with NMC-700 implies the content of graphitic carbon increases gradually because the higher carbonized temperature forces the departure of incorporated heteroatoms and the rigor of carbon matrix. The superposition peak of (100) and (101) at around 44° reflects the graphite structure [65]. Comparing the intensity of the only two peaks presented in NMC replicas, there is a slight increase synchronized with the temperature, which suggests the content of graphitic carbon as contribution in electrode improves [66].

**Figure 3:**
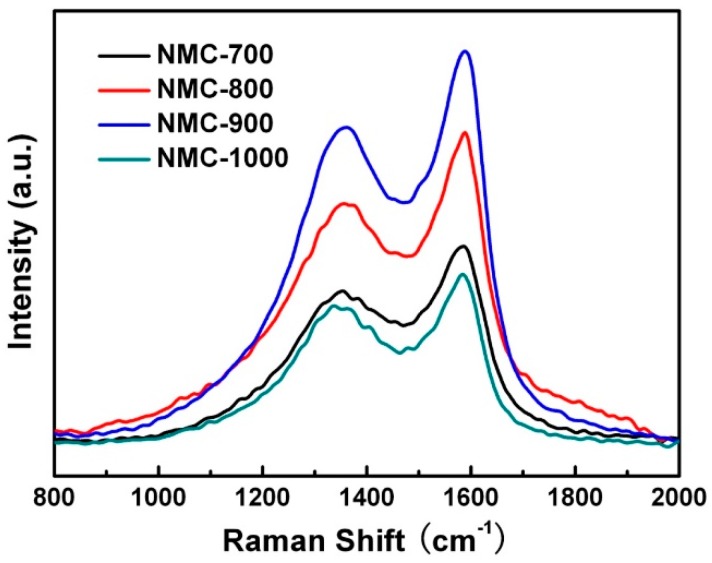
Raman spectrum of NMC in different temperatures.

Indeed, the same result has been testified by the Raman spectra shown in Figure 3. As presented in the spectrum of NMC materials, the typical G band at 1580 cm^−1^ and D band at 1350 cm^−1^ of carbon materials [67] indicate the PANI starts to carbonize to form net-frameworks at around 700 °C. Regarding the NMC materials, the ratio of *I*_G_/*I*_D_ increases with the carbonized temperature from 700 to 1000 °C, which implies that higher carbonized temperature reduce the content of defects caused by the higher diffusion rate of carbon atoms [68]. Another method adopted to illustrate the concentration of defects and the crystallinity of the carbon materials is the full width half maximum (FWHM) of D band [68]. As shown in Figure 3, the FWHM of NMC materials decreases with increasing carbonized temperatures, indicating that the pre-carbonized NMC replicas possess better crystallinity and lower content of defects at higher temperature. The electronic conductivity and corrosion resistance can be enhanced through the graphitization of carbon during electro-catalysis, and the increase of catalytically active sites of carbon materials has an important relationship with the type and content of doping heteroatoms [69,70].

**Figure 4:**
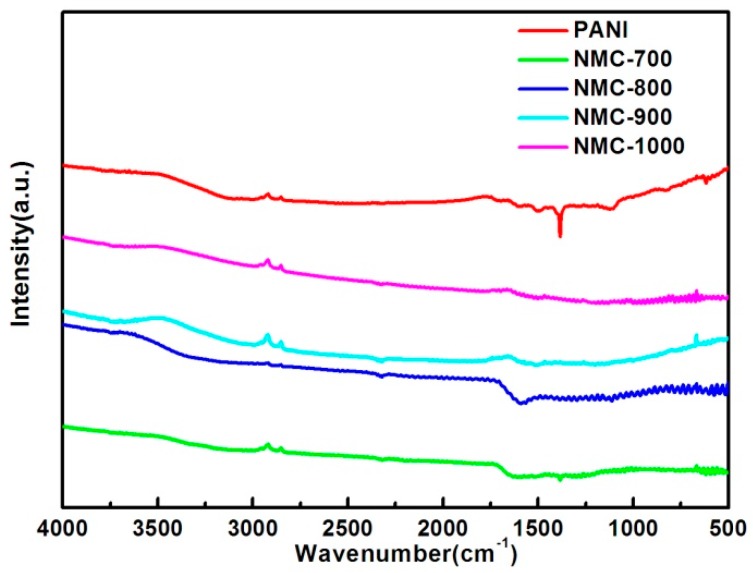
FT-IR of NMC materials carbonized at different temperatures.

In order to eliminate the influence of the silica, FT-IR is adopted to ensure no residue of the templates. As proven by the disappearance of the peak at 1100 cm^−1^ fitting Si–O–Si vibration band in the FT-IR spectrum of NMC-700 to NMC-1000 shown in Figure 4, multiporous silica bead template has been completely corroded after the dissolution by aqueous NH_4_HF_2_ solution. It also shows the characteristic peaks in PANI have almost vanished in NMC-X materials spectrum, which gives evidence of the full carbonization, and that higher carbonized temperature reduce the existence of N-containing groups and other organic groups [44]. 

**Figure 5:**
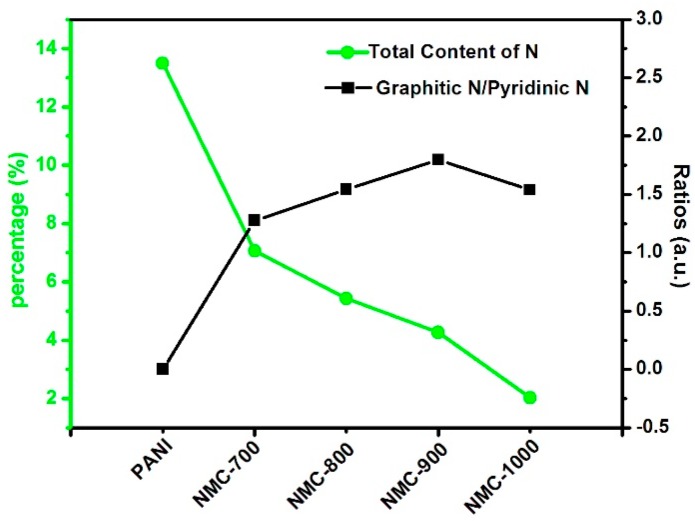
Ratio change of graphitic-N/pyridinic-N and total N contents of different samples.

N atoms in NMC play important roles in infiltration degree, conductivity and pseudocapacitance. Nitrogen content of the NMC replicas is tested by elemental analysis, as shown in Figure 5 and Table S1. The NMC samples have high and analogous amounts of nitrogen, i.e., 2.0–7.5 wt %, which is less than the content of PANI due to the carbonization. This result indicates that the N species leave and those remaining become stable gradually along with the carbonized temperature, which may be a method to control the content of N in carbon materials.

X-ray photoelectron spectroscopy (XPS) is adopted to clarify the species of carbon and nitrogen introduced onto the carbon surface, and further understand the function of nitrogen heteroatoms on capacitive performance. As shown in Figure S1, the peaks at 284.2, 398.9, and 531.6 eV can be associated to C1s, N1s, and O1s, respectively [71]. The influencing factors on the SCs electrode or the catalytic activity are not only determined by the content of N, but also by the type of N species [72,73]. Figure S1 shows the nature of the C species and the N species in NMC-800 replicas; the spectrum provides evidence that the N atoms have been successfully immigrated into the carbon matrix. The spectra of the C1s regions are shown in Figure S2, where the binding energy of the C1s group can be decomposed into six different species by deconvolution, attributed to the carbon atoms in the graphitic rings (C1 for the C=C and C2 for the C–C) and the carbons (C3 for the C–O or C–N, C4 for the carbonyl C=O, and C5 for the carboxyl O–C=O) bonded to the positively charged nitrogen that are oxidized, and recommended as charge carriers in hetero-doped carbon materials [74].

**Figure 6:**
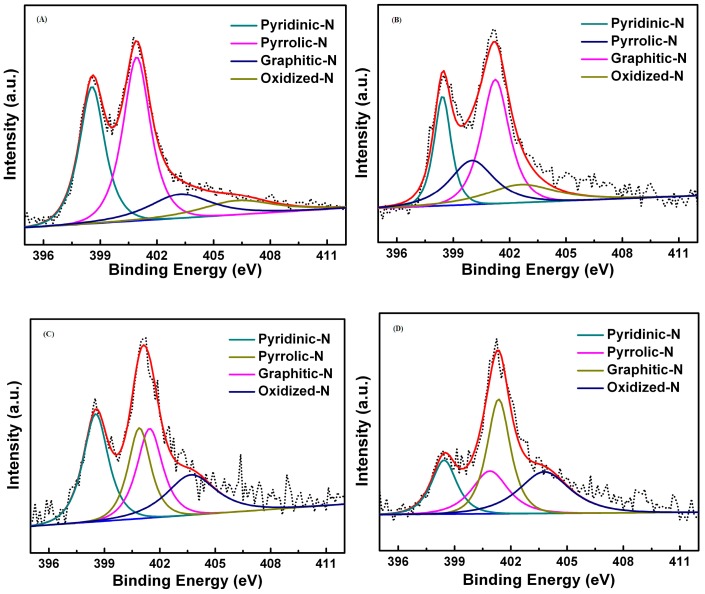
High-resolution N 1 s XPS spectra of NMC in different temperatures: (**A**) NMC-700; (**B**) NMC-800; (**C**) NMC-900; and (**D**) NMC-1000. The dotted curve is the experimental points and the red curve is the fitting curve.

Figure 6 shows N1s spectrum of NMC-800 replicas at different carbonized temperatures; four kinds of N species are examined by deconvolution: pyridinic-N (N-6, ~398.4 ± 0.2 eV), pyrrolic-N (N-5, ~400.4 ± 0.2 eV), quaternary (graphic)-N (N-Q, ~401.2 ± 0.2 eV) and pyridinic-*N*-oxide (N-X, ~403.2 ± 0.5 eV) [75]. Contrary to ORR, they are all proposed as functional sites in SCs: N-6 and N-5 located at the edges of the carbon layers make contributions to the improvement of pseudocapacitance effect; and N-Q and N-X containing the positive charge increase the electrical conductivity of electrode in SCs [76]. Obviously, the shape of the spectrum and the relative content of the main contributors change with carbonized temperature. The oxygen contained in NMC-800 replicas (Figure S1) might be rooted in the slight oxidation of the as-prepared NMC-800 replicas; previous research indicates that not only nitrogen containing functional groups in carbon materials, but also oxygen functional groups can contribute to the addition of pseudocapacitance considerably. In addition, the carbons doped with heteroatom can improve infiltration degree as well as electrode/electrolyte contact area and increase chemically reactive sites [77].

### 3.2. Structures of NMC Replicas

**Figure 7:**
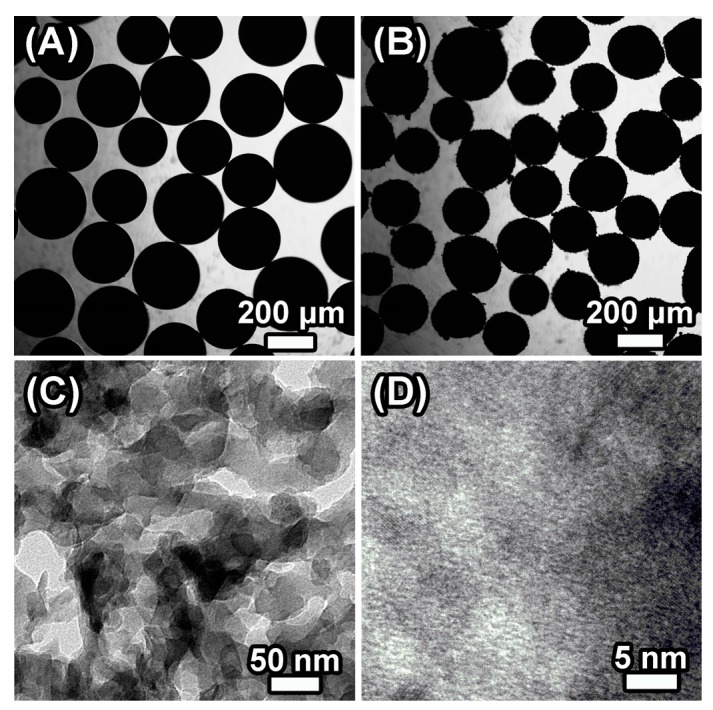
Morphology of the samples: (**A**) optical microscope of multiporous silica bead; (**B**) optical microscope of NMC-800 bead; (**C**) TEM of the NMC-800 bead; and (**D**) High-resolution transmission electron microscopy (HRTEM) of NMC-800 bead.

Optical microscope images of the NMC-800 replicas demonstrate its macroscopic structure. As shown in Figure 7A,B, the size of NMC-800 beads is similar to the silica beads, 150 to 300 μm. The NMC replicas together with the IRA-900 resin beads contain a large amount of continuous macropores and large-scale mesopores, which play important roles in promoting the mass transfer and impregnation of electrolyte, thereby the effective specific surface area increases. The internal morphology of NMC-800 (Figure 7C,D) is further investigated by TEM to reveal the macroporous structure and arrangement. The presence of abundant sponge-like meso- or macropores ranging from 20 to 100 nm is seen clearly in the TEM image. Note that the meso- or macropores partially exist and open onto the surface of NMC as shown in SEM images (Figure S3), leading to the formation of a desired 3D interconnected and continuous pore system. The hierarchical porous system can increase the contact area for fast interfacial charge transfer and mass transfer, shorten the distance for charge transfer, and increase the infiltration degree, which will enhance the capacitance behavior.

**Figure 8:**
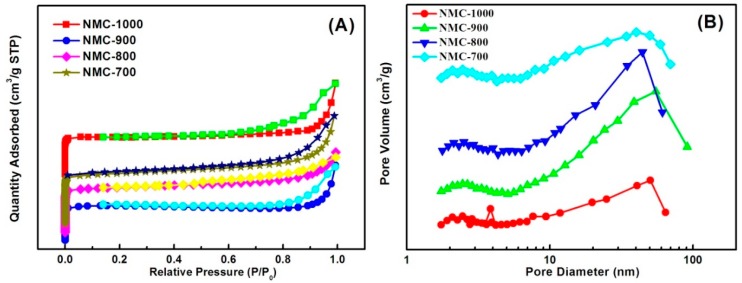
(**A**) N_2_ adsorption–desorption isotherms of NMC materials replicas, the four noted curves are adsorption isotherms and the other four curves are desorption isotherms; (**B**) the corresponding pore size distribution curves.

Figure 8A shows the nitrogen adsorption–desorption isotherms of NMC replicas. The original IRA-900 cannot be tested because of its resin property. The silica beads show a hysteresis loop from 0.5 to 1.0 at relative pressure (Figure S4A), suggesting the porosity of silica beads consists of large size mesopores and macropores, indeed, as shown in pore size distributions (PSDs), the silica beads display about 15 to 30 nm (Figure S4B). For another, the NMC materials show a step from 0.65 to 1.0 at relative pressure, indicating PSDs centered at a larger size, exactly corresponding to the PSDs displayed in Figure 8B. It is worth noting that there is a sharp rising trend in less than 0.1 relative pressure, proving the existence of micropores generated by carbonization. The isotherms illustrate BET, reflecting the specific surface area increases with temperature from 700 to 800 °C accompanied with conversion degree from PANI to carbon while many defects and pores form, with a slight reduction at 900 °C, and a sharp decrease at 1000 °C. The reason for this phenomenon is the carbonized degree is too high to allow the micropores and defects to exist. A higher specific surface value may not be the most crucial factor on the specific capacitance: if the electrolyte infiltrate on the surface of electrode unsmoothly caused by the lack of diffusion channels, the effective specific area will decrease, and the material will receive a high equivalent series resistance, which is a shortcoming of NMC materials replicas that continuous channels can solve.

### 3.3. Electrochemitry of NMC Replicas

**Figure 9:**
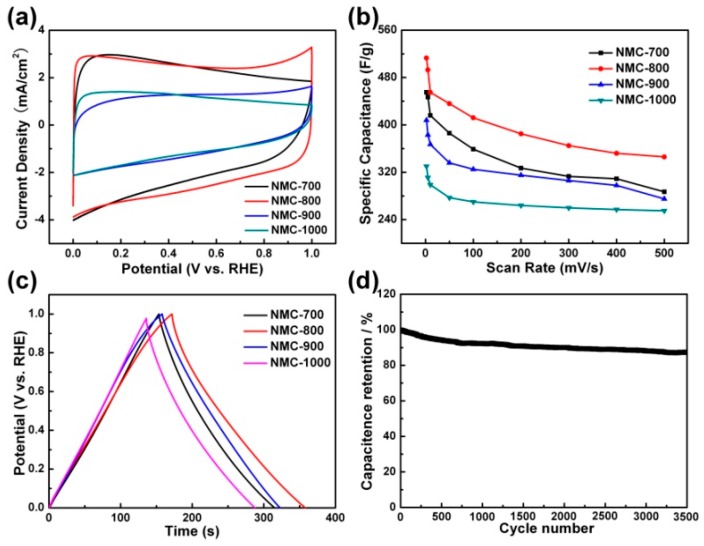
(**a**) CV curves of NMC at a scan rate of 50 mV·s^−1^; (**b**) specific capacitances of NMC at various scan rates; (**c**) galvanostatic charge/discharge curves of NMC at current densities of 1 A⋅g^−1^; and (**d**) the cycle performance of NMC-800.

Based on the high infiltration degree, high effective specific surface area and high conductivity, N-doped carbon materials are potential electrode materials for supercapacitors [45]. Following the test method of the materials as electrode of supercapacitors mentioned above, we performed the CV measurements of as-synthesized NMC materials at a scan rate from 2 to 500 mV⋅s^−1^, and the choice of voltage window is according to previously reported work [78,79]. As shown in Figure S6, the current density increases gradually with scan rate, which provides evidence that the as-prepared NMC materials possess the desirable fast charge/discharge capability for power devices. In a refining analysis, compared with NMC-800, NMC-700, NMC-900 and NMC-1000 display small integrated areas leading to lower capacitances, however, no obvious rectangular shape can be observed from their CV curves, indicating the combination of pseudocapacitance and electric double layer capacitance (EDLC) behavior. To our surprise, the CV curves of NMC-800 show nearly rectangular shapes without any redox peaks, even at high scan rates, coresponding to EDLC behavior. Moreover, the CV curve of NMC-800 owns the largest integrated area in Figure 9a, demonstrating the highest capacitance. Figure 9b exhibits the capacitance of NMC as a function of scan rate by integrating the voltammetric charge in the CV profiles. After being calculated, the specific capacitance of NMC-800 (413 F⋅g^−1^) is always higher than those of the other samples (e.g., 355 F⋅g^−1^ for NMC-700, 388 F⋅g^−1^ for NMC-900, and 270 F⋅g^−1^ for NMC-1000) at the same scan rate of 2 mV⋅s^−1^, and the value of mass capacitance is 346 F⋅g^−1^ even at a high scan rate of 500 mV⋅s^−1^, and the capacitance of other NMCs still remain over 255 F⋅g^−1^ at high scan rate of 500 mV⋅s^−1^, which is superior to the values of other reported carbon materials [45,80,81,82].

Galvanostatic charge/discharge curves as a function of current densities from 0.5 to 5 A⋅g^−1^ are measured to further estimate the performance of NMC. In contrast, even at a high current density of 5 A⋅g^−1^, all the galvanostatic charge/discharge profiles of NMC materials are nearly linear and generally symmetrical without obvious IR drop, which prove NMC materials possess a good rate capability, excellent reversibility and high Coulombic efficiency (Figure S5) [8,79]. The characteristic galvanostatic charge/discharge curves of the as-prepared materials at a given current density of 1 A⋅g^−1^ are presented in Figure 9c, indicating the ideal capacitive characteristics with a rapid I–V response. NMC-800 displays the longest discharging time and possesses the highest specific capacitance. This finding is self-consistent with the results of CV curves. The cyclic stability of the NMC-800 was examined by means of continuous CV cycling experiments. As shown in Figure 9d, evaluated by CV measurement at a scan rate 100 mV⋅s^−1^ in a 6 M KOH electrolyte, the NMC-800 presents good capacitance retention. NMC-800 exhibits a high stability of 87% capacitance retention after 3500 cycles, which shows excellent potential for practical applications as electrode materials in supercapacitors.

**Figure 10:**
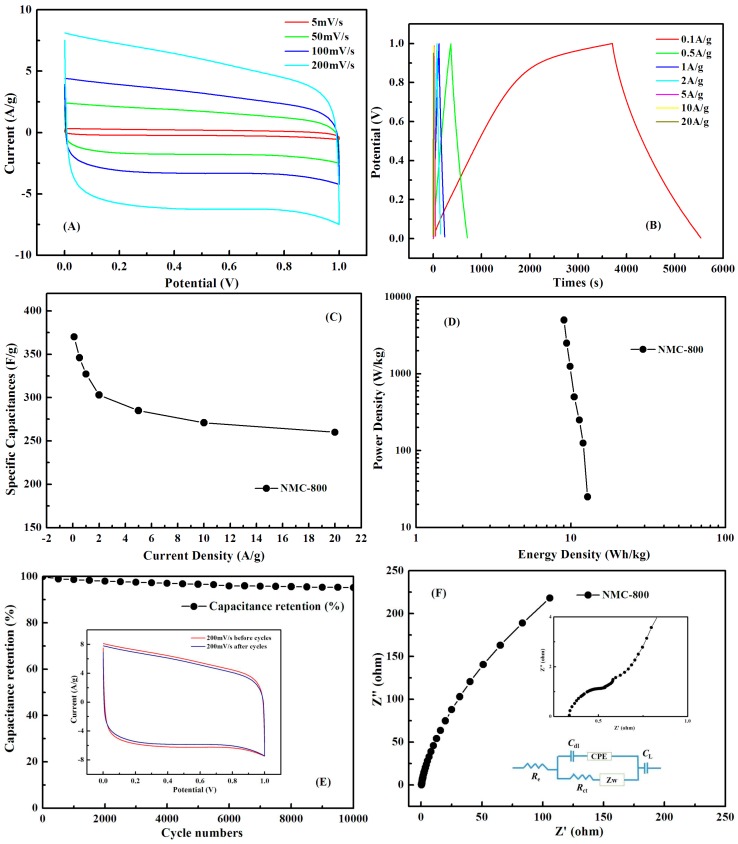
(**A**) CV curves of NMC-800 in different scan rate; (**B**) CP curves at different current densities; (**C**) specific capacitances of NMC-800 at different charge–discharge current densities; (**D**) Ragone plots; (**E**) cycling stability of the NMC-800 at 5 A·g^−1^ (inset graph is the comparison of the 1st and 10,000th CV curves); and (**F**) Nyquist plots (inset magnifies the high-frequency range and graph is the modeled equivalent circuit).

The two-electrode supercapacitor with symmetric electrodes in button cell type was also assembled to measure the electrochemical performance of NMC-800 electrodes in 6 M KOH for practical application. The cyclic voltammograms of as-assembled button cell was measured at different scan rates from 5 to 200 mV·s^−1^, the CV curves of the supercapacitor exhibited in Figure 10A are rectangular in shape, indicating ideal EDLC behavior and good reversibility. Moreover, even at 200 mV·s^−1^, the CV curves remain a nearly rectangular shape all the same, which suggests the symmetric electrodes of supercapacitor in the button cell possesses splendid rate capability and ideal electrochemical capacitive behavior for electrolyte ions diffusing rapidly to the interface of the electrode. Galvanostatic charge/discharge curves of the asymmetric electrodes in button cell were recorded with current densities from 0.1 to 20 A·g^−1^ to further evaluate the electrochemical performance (Figure 10B). A high capacitance of 327 F·g^−1^ was observed at 1 A·g^−1^, with only 19% decrease of capacitance (260 F·g^−1^) at 20 A·g^−1^. The correlation between the discharge capacitance and the various current densities for different electrodes is presented in Figure 10C. The excellent capacitance of NMC-800 should be attributed to effective Nitrogen doping and multilevel pores and channels, which enhance the mass transfer and penetration of electrolyte and charges. Figure 10D shows the Ragone plot related to energy and power densities of the NMC-800 symmetric button cell calculated from discharging curves at the different currents density shows that the highest energy density is 12.85 W·h·kg^−1^ at a power density of 25 W·kg^−1^ and remains at 9.03 W·h·kg^−1^ at a power density of 5 kW·kg^−1^, At common current density 1 A·g^−1^, the energy density is 11.35 W·h·kg^−1^ at a power density of 250 W·kg^−1^. NMC-800 material displays an excellent cyclability and capacitance retention of 95% after 10,000 charge–discharge cycles at a current density of 5 A·g^−1^ (Figure 10E), which is comparable to most carbons obtained from polymer pyrolysis [23,83,84,85,86,87,88,89,90,91,92,93,94,95].

Electrochemical impedance spectroscopy (EIS) analysis was adopted to gain a deep insight into the resistance and capacitive behavior of the symmetric electrodes in the button cell. Figure 10F shows a Nyquist plot of NMC-800 button cell with a small semicircle shape in the high-frequency region (inset of Figure 10F) and a rising curve bigger than 45° in the low-frequency region, indicating a low charge transfer resistance in electrochemical system and a superior capacitive behavior with low diffusion resistance. The EIS indicates the low equivalent series resistance (ESR) of 0.49 Ω, which contribute little to the resistive impedance of the capacitance. Furthermore, the semicircle with a small semidiameter in the high frequency region reveals the doping of nitrogen functionalities of the pyridinic and pyrrolic type and oxygen functionalities of the quinone–hydroquinone type in the charge transfer reactions. From the Nyquist plot, a corresponding equivalent circuit is shown in the inset of Figure 10F, where *R*_e_ is a combined ohmic resistance of the electrolyte and the internal resistance of the electrode, while *R*_ct_ stands for the charge transfer resistance caused by Faradaic reaction. The transition from the semicircle to the vertical curve is the Warburg resistance (*Z*_w_), which is caused by the diffusing and transporting frequency of ion in the electrolyte to the electrode surface. CPE stands for the constant phase element, *C*_dl_ is the double-layer capacitance, and *C*_L_ is the limit capacitance [96]. 

## 4. Conclusions

In conclusion, we demonstrate a new way to synthesize N-doped macroporous carbon materials using multiporous silica beads as templates. The pores of NMC replicas are composed of the micro-, large-scale meso- and macropores. Because of this hierarchical porous structure, the NMC has relatively large BET specific surface area. Combined with the XPS and Raman analyses, electrochemical tests demonstrate NMC possesses superior capacitance behavior determined by the content and relationship of graphitic N, pyrrolic N, pyridinic N and oxydic N. Accompanied with the TEM and PSDs, we may infer the hierarchical porous structures play an important role in infiltration of N atoms. This study may imply the capacitivity of SCs is cross-influenced by the content and species of N and the size and effective content of channels and hierarchical pores, while, at the same time, provides a way to synthesize macroporous materials with continuous channels, which can not only be used as electrode but also in some catalytic fields.

## Supplementary Materials

The following are available online at www.mdpi.com/2073-4360/9/1/2/s1. Table S1: Element content of C, O, N and BET and PSDs of NMC materials; Figure S1: The XPS of NMC in different temperatures; Figure S2: High-resolution C 1s XPS spectra of NMC replicas carbonized in different temperatures (A) NMC-700; (B) NMC-800; (C) NMC-900; (D) NMC-1000. The dotted curve is the experimental points and the red curve is the fitting curve; Figure S3: SEM of NMC-80; Figure S4: N_2_ adsorption-desorption isotherms of Silica beads, (A) the red curve is adsorption isotherm and the green curve is desorption isotherm. The corresponding pore size distribution curves (B); Figure S5: The CV curves of NMC-700 (a); NMC-800 (b); NMC-900 (c); NMC-1000 (d) at different scan rates; Figure S6: Galvanostatic charge/discharge curves of NMC at different current density, (a) NMC-700; (b) NMC-800; (c) NMC-900; (d) NMC-1000.

## References

[1] Li Q., Wu L., Wu G., Su D., Lv H., Zhang S., Zhu W., Casimir A., Zhu H., Mendoza-Garcia A. (2015). New approach to fully ordered FCT-FEPT nanoparticles for much enhanced electrocatalysis in acid. Nano Lett..

[2] Chen L.F., Zhang X.D., Liang H.W., Kong M., Guan Q.F., Chen P., Wu Z.Y., Yu S.H. (2012). Synthesis of nitrogen-doped porous carbon nanofibers as an efficient electrode material for supercapacitors. ACS Nano.

[3] Conway B.E. (1991). Transition from supercapacitor to battery behavior in electrochemical energy-storage. J. Electrochem. Soc..

[4] Zhu J.X., Huang L., Xiao Y.X., Shen L., Chen Q., Shi W.Z. (2014). Hydrogenated coox nanowire@NiOH_2_ nanosheet core-shell nanostructures for high-performance asymmetric supercapacitors. Nanoscale.

[5] Xu K.B., Li W.Y., Liu Q., Li B., Liu X.J., An L., Chen Z.G., Zou R.J., Hu J.Q. (2014). Hierarchical mesoporous NiCO_2_O_4_@MnO_2_ core-shell nanowire arrays on nickel foam for aqueous asymmetric supercapacitors. J. Mater. Chem. A.

[6] Morishita T., Tsumura T., Toyoda M., Przepiorski J., Morawski A.W., Konno H., Inagaki M. (2010). A review of the control of pore structure in MgO-templated nanoporous carbons. Carbon.

[7] Jiang H., Lee P.S., Li C.Z. (2013). 3D carbon based nanostructures for advanced supercapacitors. Energy Environ. Sci..

[8] Niu Z., Liu L., Zhang L., Shao Q., Zhou W., Chen X., Xie S. (2014). A universal strategy to prepare functional porous graphene hybrid architectures. Adv. Mater..

[9] Zhai Y.P., Dou Y.Q., Zhao D.Y., Fulvio P.F., Mayes R.T., Dai S. (2011). Carbon materials for chemical capacitive energy storage. Adv. Mater..

[10] Ai K., Liu Y., Ruan C., Lu L., Lu G.M. (2013). Sp2 C-dominant N-doped carbon sub-micrometer spheres with a tunable size: A versatile platform for highly efficient oxygen-reduction catalysts. Adv. Mater..

[11] Ma Y., Sun L., Huang W., Zhang L., Zhao J., Fan Q., Huang W. (2011). Three-dimensional nitrogen-doped carbon nanotubes/graphene structure used as a metal-free electrocatalyst for the oxygen reduction reaction. J. Phys. Chem. C.

[12] Li Y., Li T.T., Yao M., Liu S.Q. (2012). Metal-free nitrogen-doped hollow carbon spheres synthesized by thermal treatment of poly(*o*-phenylenediamine) for oxygen reduction reaction in direct methanol fuel cell applications. J. Mater. Chem..

[13] Lin Z., Waller G.H., Liu Y., Liu M., Wong C.-P. (2013). Simple preparation of nanoporous few-layer nitrogen-doped graphene for use as an efficient electrocatalyst for oxygen reduction and oxygen evolution reactions. Carbon.

[14] Sun Y., Li C., Shi G. (2012). Nanoporous nitrogen doped carbon modified graphene as electrocatalyst for oxygen reduction reaction. J. Mater. Chem..

[15] Chen W.-M., Qie L., Shen Y., Sun Y.-M., Yuan L.-X., Hu X.-L., Zhang W.-X., Huang Y.-H. (2013). Superior lithium storage performance in nanoscaled MnO promoted by N-doped carbon webs. Nano Energy.

[16] Li H., Shen L., Zhang X., Wang J., Nie P., Che Q., Ding B. (2013). Nitrogen-doped carbon coated Li_4_Ti_5_O_12_ nanocomposite: Superior anode materials for rechargeable lithium ion batteries. J. Power Sources.

[17] Qiao Y., Hu X., Liu Y., Chen C., Xu H., Hou D., Hu P., Huang Y. (2013). Conformal N-doped carbon on nanoporous TiO_2_ spheres as a high-performance anode material for lithium-ion batteries. J. Mater. Chem. A.

[18] Tan L., Pan L., Cao C., Wang B., Li L. (2014). Nitrogen-doped carbon coated TiO_2_ nanocomposites as anode material to improve cycle life for lithium-ion batteries. J. Power Sources.

[19] Candelaria S.L., Garcia B.B., Liu D., Cao G. (2012). Nitrogen modification of highly porous carbon for improved supercapacitor performance. J. Mater. Chem..

[20] Lai L., Wang L., Yang H., Sahoo N.G., Tam Q.X., Liu J., Poh C.K., Lim S.H., Shen Z., Lin J. (2012). Tuning graphene surface chemistry to prepare graphene/polypyrrole supercapacitors with improved performance. Nano Energy.

[21] Hulicova-Jurcakova D., Kodama M., Shiraishi S., Hatori H., Zhu Z.H., Lu G.Q. (2009). Nitrogen-enriched nonporous carbon electrodes with extraordinary supercapacitance. Adv. Funct. Mater..

[22] Zhao J., Lai H.-W., Lyu Z.-Y., Jiang Y.-F., Xie K., Wang X.-Z., Wu Q., Yang L.-J., Jin Z., Ma Y.W. (2015). Hydrophilic hierarchical nitrogen-doped carbon nanocages for ultrahigh supercapacitive performance. Adv. Mater..

[23] Chen P., Yang J.-J., Li S.-S., Wang Z., Xiao T.-Y., Qian Y.-H., Yu S.-H. (2013). Hydrothermal synthesis of macroscopic nitrogen-doped graphene hydrogels for ultrafast supercapacitor. Nano Energy.

[24] Zhu H., Wang X.L., Liu X.X., Yang X.R. (2012). Integrated synthesis of poly(*o*-phenylenediamine)-derived carbon materials for high performance supercapacitors. Adv. Mater..

[25] Lee W.H., Moon J.H. (2014). Monodispersed N-doped carbon nanospheres for supercapacitor application. ACS Appl. Mater. Interfaces.

[26] Li Y., Dong J., Zhang J., Zhao X., Yu P., Jin L., Zhang Q. (2015). Nitrogen-doped carbon membrane derived from polyimide as free-standing electrodes for flexible supercapacitors. Small.

[27] Li Q., Cao R., Cho J., Wu G. (2014). Nanocarbon electrocatalysts for oxygen reduction in alkaline media for advanced energy conversion and storage. Adv. Energy Mater..

[28] Shen W., Fan W. (2013). Nitrogen-containing porous carbons: Synthesis and application. J. Mater. Chem. A.

[29] Zheng Y., Jiao Y., Chen J., Liu J., Liang J., Du A., Zhang W., Zhu Z., Smith S.C., Jaroniec M. (2011). Nanoporous graphitic-C_3_N_4_@ carbon metal-free electrocatalysts for highly efficient oxygen reduction. J. Am. Chem. Soc..

[30] Jiang Y., Lu Y., Wang X., Bao Y., Chen W., Niu L. (2014). A cobalt–nitrogen complex on N-doped three-dimensional graphene framework as a highly efficient electrocatalyst for oxygen reduction reaction. Nanoscale.

[31] Zhang Y., Jiang W.-J., Zhang X., Guo L., Hu J.-S., Wei Z., Wan L.-J. (2014). Engineering self-assembled N-doped graphene–carbon nanotube composites towards efficient oxygen reduction electrocatalysts. Phys. Chem. Chem. Phys..

[32] Yoon S., Liao C., Sun X.G., Bridges C.A., Unocic R.R., Nanda J., Dai S., Paranthaman M.P. (2012). Conductive surface modification of lifepo4 with nitrogen-doped carbon layers for lithium-ion batteries. J. Mater. Chem..

[33] Zhou Y.K., Neyerlin K., Olson T.S., Pylypenko S., Bult J., Dinh H.N., Gennett T., Shao Z.P., O’Hayre R. (2010). Enhancement of Pt and Pt-alloy fuel cell catalyst activity and durability via nitrogen-modified carbon supports. Energy Environ. Sci..

[34] Paraknowitsch J.P., Zhang J., Su D.S., Thomas A., Antonietti M. (2010). Ionic liquids as precursors for nitrogen-doped graphitic carbon. Adv. Mater..

[35] Vujkovic M., Gavrilov N., Pasti I., Krstic J., Travas-Sejdic J., Ciric-Marjanovic G., Mentus S. (2013). Superior capacitive and electrocatalytic properties of carbonized nanostructured polyaniline upon a low-temperature hydrothermal treatment. Carbon.

[36] Li Z., Zhang L., Amirkhiz B.S., Tan X.H., Xu Z.W., Wang H.L., Olsen B.C., Holt C.M.B., Mitlin D. (2012). Carbonized chicken eggshell membranes with 3D architectures as high-performance electrode materials for supercapacitors. Adv. Energy Mater..

[37] Ma T.Y., Dai S., Jaroniec M., Qiao S.Z. (2014). Graphitic carbon nitride nanosheet-carbon nanotube three-dimensional porous composites as high-performance oxygen evolution electrocatalysts. Angew. Chem. Int. Ed..

[38] Tian G.L., Zhang Q., Zhang B.S., Jin Y.G., Huang J.Q., Su D.S., Wei F. (2014). Toward full exposure of “active sites”: Nanocarbon electrocatalyst with surface enriched nitrogen for superior oxygen reduction and evolution reactivity. Adv. Funct. Mater..

[39] Wang X., Liu Y., Zhu D., Zhang L., Ma H., Yao N., Zhang B. (2002). Controllable growth, structure, and low field emission of well-aligned Cn x nanotubes. J. Phys. Chem. B.

[40] Gong K., Du F., Xia Z., Durstock M., Dai L. (2009). Nitrogen-doped carbon nanotube arrays with high electrocatalytic activity for oxygen reduction. Science.

[41] Duan X., Ao Z., Sun H., Indrawirawan S., Wang Y., Kang J., Liang F., Zhu Z.H., Wang S. (2015). Nitrogen-doped graphene for generation and evolution of reactive radicals by metal-free catalysis. ACS Appl. Mater. Interfaces.

[42] Chien C.T., Hiralal P., Wang D.Y., Huang I.S., Chen C.C., Chen C.W., Amaratunga G.A.J. (2015). Graphene-based integrated photovoltaic energy harvesting/storage device. Small.

[43] Reddy A.L.M., Srivastava A., Gowda S.R., Gullapalli H., Dubey M., Ajayan P.M. (2010). Synthesis of nitrogen-doped graphene films for lithium battery application. ACS Nano.

[44] He W., Jiang C., Wang J., Lu L. (2014). High-rate oxygen electroreduction over graphitic-N species exposed on 3D hierarchically porous nitrogen-doped carbons. Angew. Chem. Int. Ed. Engl..

[45] Wei J., Zhou D.D., Sun Z.K., Deng Y.H., Xia Y.Y., Zhao D.Y. (2013). A controllable synthesis of rich nitrogen-doped ordered mesoporous carbon for CO_2_ capture and supercapacitors. Adv. Funct. Mater..

[46] Kang K.Y., Lee B.I., Lee J.S. (2009). Hydrogen adsorption on nitrogen-doped carbon xerogels. Carbon.

[47] Raymundo-Pinero E., Cazorla-Amoros D., Linares-Solano A. (2003). The role of different nitrogen functional groups on the removal of SO_2_ from flue gases by N-doped activated carbon powders and fibres. Carbon.

[48] Yang C.-M., Kaneko K. (2002). Nitrogen-doped activated carbon fiber as an applicant for no adsorbent. J. Colloid Interface Sci..

[49] Matter P.H., Zhang L., Ozkan U.S. (2006). The role of nanostructure in nitrogen-containing carbon catalysts for the oxygen reduction reaction. J. Catal..

[50] Ra E.J., Raymundo-Pinero E., Lee Y.H., Beguin F. (2009). High power supercapacitors using polyacrylonitrile-based carbon nanofiber paper. Carbon.

[51] Kim N.D., Kim W., Joo J.B., Oh S., Kim P., Kim Y., Yi J. (2008). Electrochemical capacitor performance of N-doped mesoporous carbons prepared by ammoxidation. J. Power Sources.

[52] Kierzek K., Frackowiak E., Lota G., Gryglewicz G., Machnikowski J. (2004). Electrochemical capacitors based on highly porous carbons prepared by KOH activation. Electrochim. Acta.

[53] Ferrero G., Fuertes A., Sevilla M. (2015). N-doped microporous carbon microspheres for high volumetric performance supercapacitors. Electrochim. Acta.

[54] Ren Y., Zhang J., Xu Q., Chen Z., Yang D., Wang B., Jiang Z. (2014). Biomass-derived three-dimensional porous N-doped carbonaceous aerogel for efficient supercapacitor electrodes. RSC Adv..

[55] White R.J., Yoshizawa N., Antonietti M., Titirici M.-M. (2011). A sustainable synthesis of nitrogen-doped carbon aerogels. Green Chem..

[56] Shi Q., Zhang R., Lv Y., Deng Y., Elzatahrya A.A., Zhao D. (2015). Nitrogen-doped ordered mesoporous carbons based on cyanamide as the dopant for supercapacitor. Carbon.

[57] Daems N., Sheng X., Vankelecom I.F.J., Pescarmona P.P. (2014). Metal-free doped carbon materials as electrocatalysts for the oxygen reduction reaction. J. Mater. Chem. A.

[58] Jeon I.Y., Yu D.S., Bae S.Y., Choi H.J., Chang D.W., Dai L.M., Baek J.B. (2011). Formation of large-area nitrogen-doped graphene film prepared from simple solution casting of edge-selectively functionalized graphite and its electrocatalytic activity. Chem. Mater..

[59] Gavrilov N., Pasti I.A., Mitric M., Travas-Sejdic J., Ciric-Marjanovic G., Mentus S.V. (2012). Electrocatalysis of oxygen reduction reaction on polyaniline-derived nitrogen-doped carbon nanoparticle surfaces in alkaline media. J. Power Sources.

[60] Liu Y.L., Shi C.X., Xu X.Y., Sun P.C., Chen T.H. (2015). Nitrogen-doped hierarchically porous carbon spheres as efficient metal-free electrocatalysts for an oxygen reduction reaction. J. Power Sources.

[61] Lin K.F., Lebedev O.I., Van Tendeloo G., Jacobs P.A., Pescarmona P.P. (2010). Titanosilicate beads with hierarchical porosity: Synthesis and application as epoxidation catalysts. Chem. Eur. J..

[62] Wu Z.S., Liu Z., Parvez K., Feng X., Mullen K. (2015). Ultrathin printable graphene supercapacitors with AC line-filtering performance. Adv. Mater..

[63] Zhang Z.M., Wan M.X., Wei Y. (2006). Highly crystalline polyaniline nanostructures doped with dicarhoxylic acids. Adv. Funct. Mater..

[64] Sevilla M., Fuertes A.B. (2006). Catalytic graphitization of templated mesoporous carbons. Carbon.

[65] Panomsuwan G., Saito N., Ishizaki T. (2015). Simple one-step synthesis of fluorine-doped carbon nanoparticles as potential alternative metal-free electrocatalysts for oxygen reduction reaction. J. Mater. Chem. A.

[66] Qie L., Chen W.M., Wang Z.H., Shao Q.G., Li X., Yuan L.X., Hu X.L., Zhang W.X., Huang Y.H. (2012). Nitrogen-doped porous carbon nanofiber webs as anodes for lithium ion batteries with a superhigh capacity and rate capability. Adv. Mater..

[67] Shin J.-K., Lee C.S., Lee K.-R., Eun K.Y. (2001). Effect of residual stress on the raman-spectrum analysis of tetrahedral amorphous carbon films. Appl. Phys. Lett..

[68] Sharifi T., Nitze F., Barzegar H.R., Tai C.-W., Mazurkiewicz M., Malolepszy A., Stobinski L., Wågberg T. (2012). Nitrogen doped multi walled carbon nanotubes produced by cvd-correlating XPS and raman spectroscopy for the study of nitrogen inclusion. Carbon.

[69] Ma F.X., Wang J., Wang F.B., Xia X.H. (2015). The room temperature electrochemical synthesis of N-doped graphene and its electrocatalytic activity for oxygen reduction. Chem. Commun..

[70] Li Y., Zhao Y., Cheng H., Hu Y., Shi G., Dai L., Qu L. (2012). Nitrogen-doped graphene quantum dots with oxygen-rich functional groups. J. Am. Chem. Soc..

[71] Li N., Wang Z.Y., Zhao K.K., Shi Z.J., Gu Z.N., Xu S.K. (2010). Large scale synthesis of N-doped multi-layered graphene sheets by simple arc-discharge method. Carbon.

[72] Horikawa T., Sakao N., Sekida T., Hayashi J., Do D.D., Katoh M. (2012). Preparation of nitrogen-doped porous carlbon by ammonia gas treatment and the effects of N-doping on water adsorption. Carbon.

[73] Hao L., Li X.L., Zhi L.J. (2013). Carbonaceous electrode materials for supercapacitors. Adv. Mater..

[74] Xu B., Zheng D.F., Jia M.Q., Cao G.P., Yang Y.S. (2013). Nitrogen-doped porous carbon simply prepared by pyrolyzing a nitrogen-containing organic salt for supercapacitors. Electrochim. Acta.

[75] He Z.W., Lu Q.F., Lin Q.L. (2013). Fabrication, characterization and application of nitrogen-containing carbon nanospheres obtained by pyrolysis of lignosulfonate/poly(2-ethylaniline). Bioresour. Technol..

[76] Hulicova-Jurcakova D., Seredych M., Lu G.Q., Bandosz T.J. (2009). Combined effect of nitrogen- and oxygen-containing functional groups of microporous activated carbon on its electrochemical performance in supercapacitors. Adv. Funct. Mater..

[77] Huang Y., Liang J.J., Chen Y.S. (2012). An overview of the applications of graphene-based materials in supercapacitors. Small.

[78] Zhang J., Chen G., Zhang Q., Kang F., You B. (2015). Self-assembly synthesis of N-doped carbon aerogels for supercapacitor and electrocatalytic oxygen reduction. ACS Appl. Mater. Interfaces.

[79] Long C., Qi D., Wei T., Yan J., Jiang L., Fan Z. (2014). Nitrogen-doped carbon networks for high energy density supercapacitors derived from polyaniline coated bacterial cellulose. Adv. Funct. Mater..

[80] Chung D.Y., Lee K.J., Yu S.H., Kim M., Lee S.Y., Kim O.H., Park H.J., Sung Y.E. (2015). Alveoli-inspired facile transport structure of N-doped porous carbon for electrochemical energy applications. Adv. Energy Mater..

[81] Hu Y.T., Liu H.J., Ke Q.Q., Wang J. (2014). Effects of nitrogen doping on supercapacitor performance of a mesoporous carbon electrode produced by a hydrothermal soft-templating process. J. Mater. Chem. A.

[82] Sun M.Q., Wang G.C., Yang C.Y., Jiang H., Li C.Z. (2015). A graphene/carbon nanotube@π-conjugated polymer nanocomposite for high-performance organic supercapacitor electrodes. J. Mater. Chem. A.

[83] Zhang L., Shi G. (2011). Preparation of highly conductive graphene hydrogels for fabricating supercapacitors with high rate capability. J. Phys. Chem. C.

[84] Yoon Y., Lee K., Baik C., Yoo H., Min M., Park Y., Lee S.M., Lee H. (2013). Anti-solvent derived non-stacked reduced graphene oxide for high performance supercapacitors. Adv. Mater..

[85] Xu Y., Lin Z., Zhong X., Huang X., Weiss N.O., Huang Y., Duan X. (2014). Holey graphene frameworks for highly efficient capacitive energy storage. Nat. Commun..

[86] Xu B., Hou S., Cao G., Wu F., Yang Y. (2012). Sustainable nitrogen-doped porous carbon with high surface areas prepared from gelatin for supercapacitors. J. Mater. Chem..

[87] Wu L., Li W., Li P., Liao S., Qiu S., Chen M., Guo Y., Li Q., Zhu C., Liu L. (2014). Powder, paper and foam of few-layer graphene prepared in high yield by electrochemical intercalation exfoliation of expanded graphite. Small.

[88] Wang Y., Shi Z., Huang Y., Ma Y., Wang C., Chen M., Chen Y. (2009). Supercapacitor devices based on graphene materials. J. Phys. Chem. C.

[89] Wang H., Sun X., Liu Z., Lei Z. (2014). Creation of nanopores on graphene planes with mgo template for preparing high-performance supercapacitor electrodes. Nanoscale.

[90] Wang C., Wang Y., Graser J., Zhao R., Gao F., O’Connell M.J. (2013). Solution-based carbohydrate synthesis of individual solid, hollow, and porous carbon nanospheres using spray pyrolysis. ACS Nano.

[91] Tan Y., Xu C., Chen G., Liu Z., Ma M., Xie Q., Zheng N., Yao S. (2013). Synthesis of ultrathin nitrogen-doped graphitic carbon nanocages as advanced electrode materials for supercapacitor. ACS Appl. Mater. Interfaces.

[92] Mao B.S., Wen Z., Bo Z., Chang J., Huang X., Chen J. (2014). Hierarchical nanohybrids with porous cnt-networks decorated crumpled graphene balls for supercapacitors. ACS Appl. Mater. Interfaces.

[93] Liu Z., Fu D., Liu F., Han G., Liu C., Chang Y., Xiao Y., Li M., Li S. (2014). Mesoporous carbon nanofibers with large cage-like pores activated by tin dioxide and their use in supercapacitor and catalyst support. Carbon.

[94] Li Y., Li Z., Shen P.K. (2013). Simultaneous formation of ultrahigh surface area and three-dimensional hierarchical porous graphene-like networks for fast and highly stable supercapacitors. Adv. Mater..

[95] Jeong H.M., Lee J.W., Shin W.H., Choi Y.J., Shin H.J., Kang J.K., Choi J.W. (2011). Nitrogen-doped graphene for high-performance ultracapacitors and the importance of nitrogen-doped sites at basal planes. Nano Lett..

[96] Chang J., Jin M., Yao F., Kim T.H., Viet Thong L., Yue H., Gunes F., Li B., Ghosh A., Xie S. (2013). Asymmetric supercapacitors based on graphene/MnO_2_ nanospheres and graphene/MoO_3_ nanosheets with high energy density. Adv. Funct. Mater..

