# Supplementary material for: Nitrogen Doped Macroporous Carbon as Electrode Materials for High Capacity of Supercapacitor
Source: Polymers (Basel). 2017 Jan 13;9(1):2. doi: 10.3390/polym9010002 (PMC6432412; doi:10.3390/polym9010002)
Supplement: Supplementary file 1 [file polymers-09-00002-s001.pdf]

Supplementary Materials: Nitrogen Doped Macroporous Carbon as Electrode Materials for High Capacity of Supercapacitor

Yudong Li, Xianzhu Xu, Yanzhen He, Yanqiu Jiang and Kaifeng Lin

Table S1. Element content of C, O, N and BET and PSDs of NMC materials.

	Elements content wt %			BET surface area ($m^2 \cdot g^{-1}$)	PSDs (nm)
	C	N	O		
NMC-700	83.1	7.07	2.74	~286	~41
NMC-800	89.3	5.43	2.02	~443	~45
NMC-900	91.2	4.27	1.97	~407	~55
NMC-1000	94.7	2.03	1.43	~129	~50

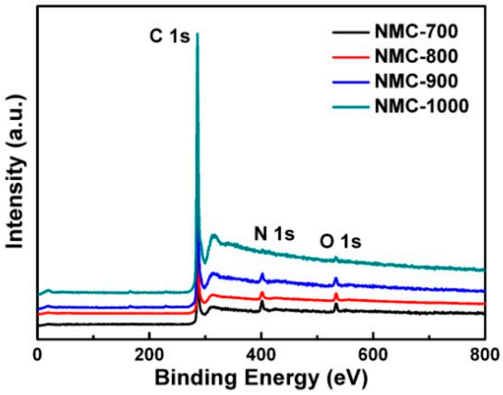

Figure S1. The XPS of NMC in different temperatures.

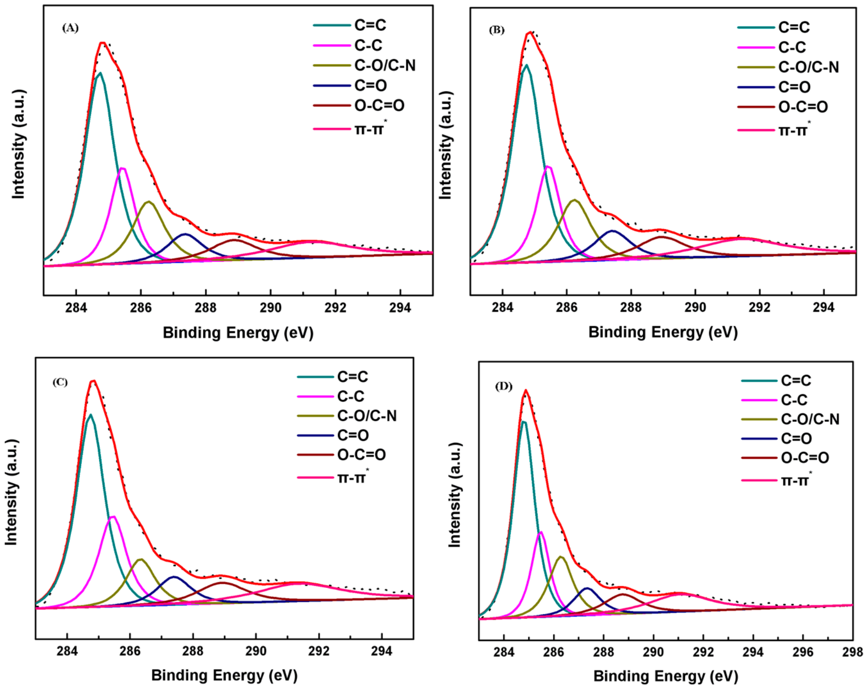

Figure S2. High-resolution C 1s XPS spectra of NMC replicas carbonized in different temperatures (**A**) NMC-700; (**B**) NMC-800; (**C**) NMC-900; (**D**) NMC-1000. The dotted curve is the experimental points and the red curve is the fitting curve.

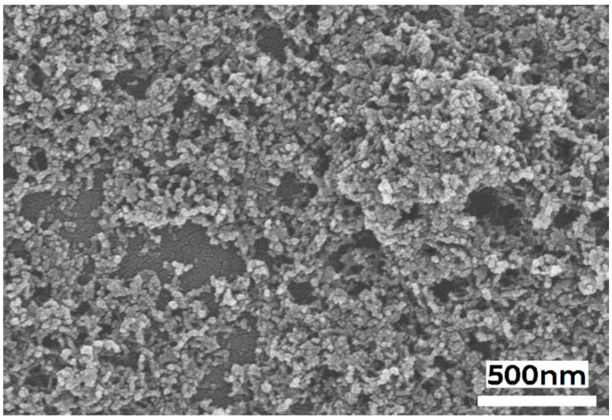

Figure S3. SEM of NMC-800.

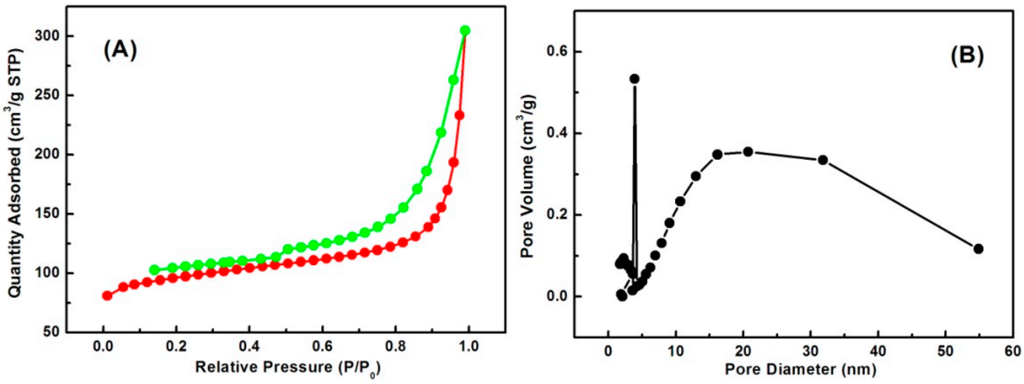

Figure S4. N_2 adsorption-desorption isotherms of Silica beads. (**A**) The red curve is adsorption isotherm and the green curve is desorption isotherm; the corresponding pore size distribution curves (**B**).

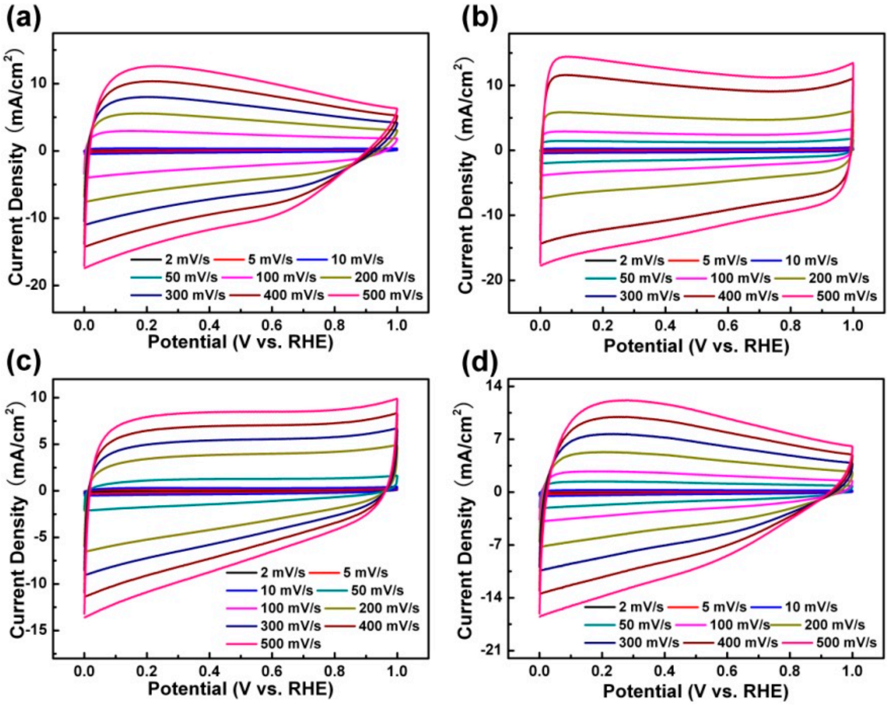

Figure S5. The CV curves of NMC-700 (**a**); NMC-800 (**b**); NMC-900 (**c**); NMC-1000 (**d**) at different scan rates.

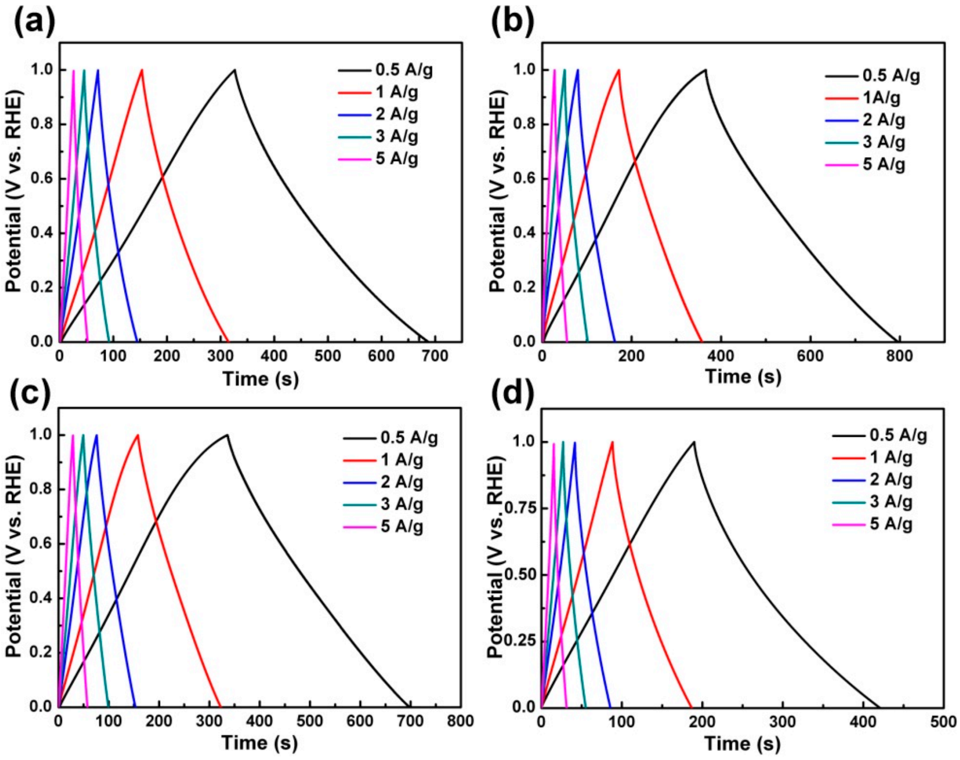

Figure S6. Galvanostatic charge/discharge curves of NMC at different current density. (**a**) NMC-700; (**b**) NMC-800; (**c**) NMC-900; (**d**) NMC-1000.
